# Novel approach to quantify mitochondrial content and intrinsic bioenergetic efficiency across organs

**DOI:** 10.1038/s41598-020-74718-1

**Published:** 2020-10-19

**Authors:** Kelsey L. McLaughlin, James T. Hagen, Hannah S. Coalson, Margaret A. M. Nelson, Kimberly A. Kew, Ashley R. Wooten, Kelsey H. Fisher-Wellman

**Affiliations:** 1 Department of Physiology, Brody School of Medicine, East Carolina University, Greenville, NC 27834 USA; 2 Department of Biochemistry and Molecular Biology, Brody School of Medicine, East Carolina University, Greenville, NC 27834 USA; 3 East Carolina Diabetes and Obesity Institute, Greenville, NC 27834 USA

**Keywords:** Energy metabolism, Proteomics

## Abstract

Human disease pathophysiology commonly involves metabolic disruption at both the cellular and subcellular levels. Isolated mitochondria are a powerful model for separating global cellular changes from intrinsic mitochondrial alterations. However, common laboratory practices for isolating mitochondria (e.g., differential centrifugation) routinely results in organelle preparations with variable mitochondrial purity. To overcome this issue, we developed a mass spectrometry-based method that quantitatively evaluates sample-specific percent mitochondrial enrichment. Sample-specific mitochondrial enrichment was then used to correct various biochemical readouts of mitochondrial function to a ‘fixed’ amount of mitochondrial protein, thus allowing for intrinsic mitochondrial bioenergetics, relative to the underlying proteome, to be assessed across multiple mouse tissues (e.g., heart, brown adipose, kidney, liver). Our results support the use of mitochondrial-targeted nLC-MS/MS as a method to quantitate mitochondrial enrichment on a per-sample basis, allowing for unbiased comparison of functional parameters between populations of mitochondria isolated from metabolically distinct tissues. This method can easily be applied across multiple experimental settings in which intrinsic shifts in the mitochondrial network are suspected of driving a given physiological or pathophysiological outcome.

## Introduction

Metabolic dysregulation has been implicated as a key component in the pathophysiology of several diseases, including diabetes, kidney disease, and cancer, among others^1–3^. Reflective of this dysregulation, increasing evidence has indicated that mitochondria, the subcellular sites of oxidative phosphorylation (OXPHOS), undergo remodeling in these disease states that may include depressed respiration, increased production of reactive oxygen species, loss of cristae, or loss of mitochondrial volume^4^. These adaptations occur in addition to the broader physiological consequences of altered metabolism such as shifts in protein expression, deposition of lipid droplets, or increased fibrosis. Together, these interconnected changes result in a tissue that is fundamentally distinct from the healthy ‘normal’ tissue. Furthering our understanding of these diseases thus relies upon comparison of ‘normal’ to ‘diseased’ metabolic states at the cellular and subcellular level, in a multitude of tissue backgrounds.

To separate global changes to metabolism from intrinsic mitochondrial remodeling, investigators commonly use isolated mitochondrial preparations obtained through differential centrifugation^5^. This simple process has been reliably implemented in a variety of tissues to produce intact, functional mitochondria for bioenergetic evaluation. For the normalization of data between isolations from the same tissue type, values are typically scaled to the amount of protein used per experiment. However, the crude mitochondrial pellet acquired through differential centrifugation will also contain non-mitochondrial contaminants including lysosomes, peroxisomes, and portions of other subcellular organelles that are of similar density to mitochondria^6–8^. Further, it is likely that contamination is not uniform across tissues, as each tissue maintains a different proportion of these organelles^9–11^, presumably to perform specialized functions. Similarly, given the cellular consequences of disease listed above, there will likely be an additional impact of disease state upon the purity of the mitochondrial preparation^12^. Accordingly, equitable comparison of mitochondrial function across different tissues, as well as between diseased/healthy states, requires reliable normalization that corrects for the mitochondrial purity across subcellular isolations.

One prevailing strategy for normalization is to estimate mitochondrial content through measuring the activity of citrate synthase (CS), an enzyme at the intersection of fuel catalysis and entry of metabolites into the citric acid cycle. Previous work has demonstrated a strong correlation between mitochondrial content and CS activity in skeletal muscle^13^. However, to our knowledge, this correlation has not been validated in other tissues, nor in different disease states, potentially limiting its application for evaluating isolation purity for all experimental models. Moreover, as the mitochondrion represents a complex collection of integrated pathways, it seems unlikely that the activity of any single enzyme would be reflective of mitochondrial content across tissues with differing energetic demands or constraints.

The present study sought to address this technical barrier inherent to quantifying inter-mitochondrial differences across organs through the use of label-free, mitochondrial-targeted nanoLC-MS/MS paired with comprehensive bioenergetic phenotyping. By carrying out quantitative proteomic screens on aliquots of mitochondria used for functional analysis, this allowed us to directly compute mitochondrial vs. non-mitochondrial protein on a per-sample basis. Such analyses generated a mitochondrial enrichment factor (MEF) that empirically reflected the mitochondrial purity of a given isolation. We subsequently used this MEF to identify potential protein biomarkers of mitochondrial content shared across tissues, as well as directly compare mitochondrial bioenergetic fluxes between tissues through differential protein expression, high resolution respirometry, and ATP production profiles. In order to provide experimental contrast, four metabolically diverse tissues were compared: brown adipose tissue, heart, kidney, and liver.

## Results

### Citrate synthase activity does not reflect mitochondrial content across distinct mouse tissues

**Figure 1:**
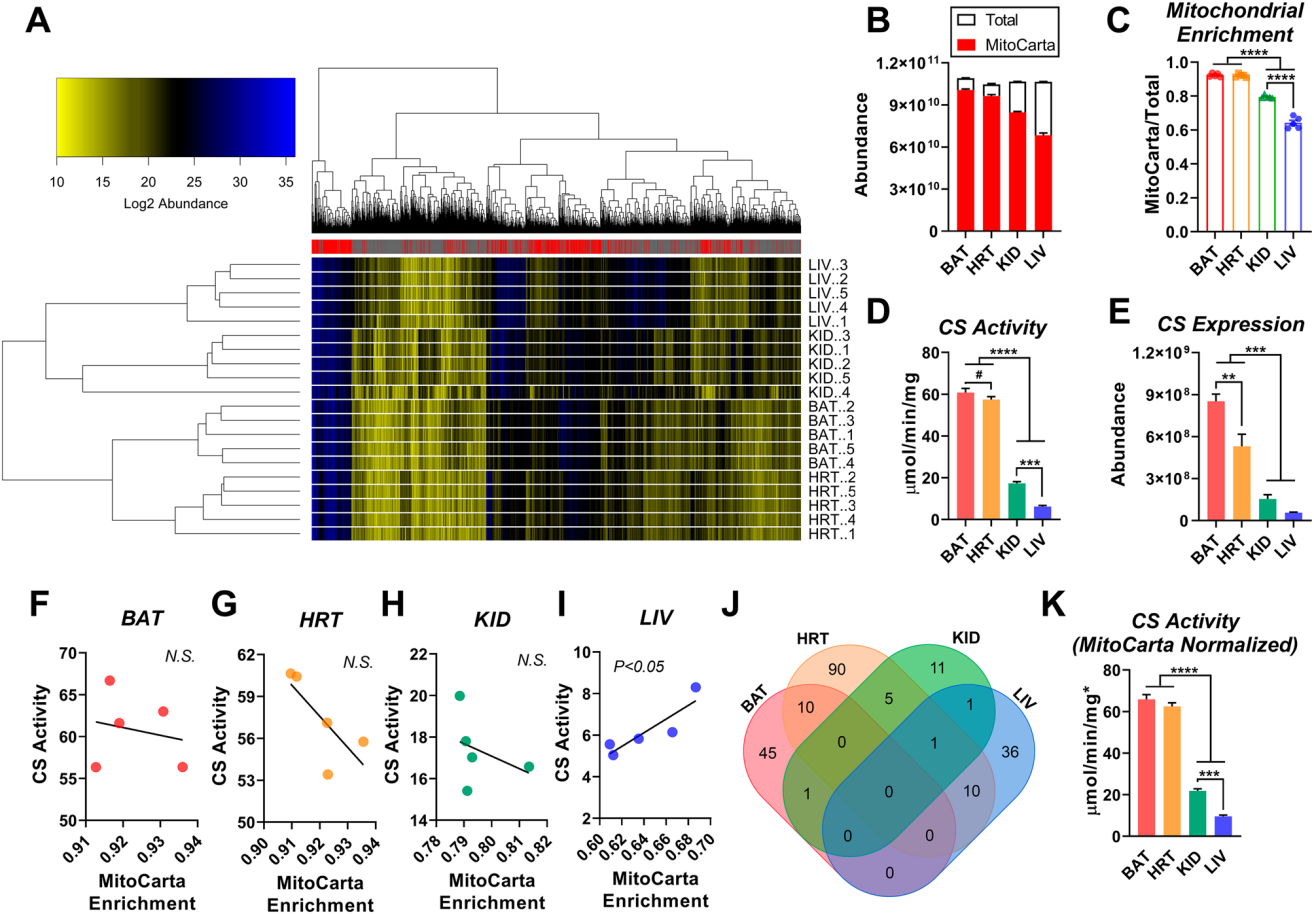
Quantification of percent mitochondrial enrichment using subcellular proteomics. (**A**) Heatmap displaying abundance (Log2) of mitochondrial and non-mitochondrial high confidence proteins from isolated mitochondria samples prepared from BAT, HRT, KID, and LIV. Mitochondrial proteins are indicated in the legend by a ‘red’ mark. Mitochondrial proteins were assigned using the MitoCarta 2.0 database. (**B**) Summed total abundance of all proteins, relative to mitochondrial proteins. (**C**) Mitochondrial enrichment factors per tissue, derived from the ratio of mitochondrial to total protein abundance. **(D)** Citrate synthase (CS) activity in isolated mitochondria, normalized to protein. (**E**) CS abundance. (**F–J**) Correlation between CS activity and MitoCarta enrichment. **(J)** Overlapping proteins per tissue that correlate with MitoCarta enrichment. **(K)** CS activity normalized to protein and corrected for each sample’s mitochondrial enrichment factor (MEF). To do this, CS activity displayed in panel (**D**) was multiplied by the sample-specific MEF. (**A**) Figure generated using R Studio version 3.6.2 (heatmap.plus). (**B–I, K**) Figures were generated using GraphPad Prism 8 software (Version 8.4.2) (**J**) Figure generated using https://bioinformatics.psb.ugent.be/webtools/Venn/. Data information: Data are presented as mean ± SEM, N = 5/group. **P < 0.01, ***P < 0.001, ****P < 0.0001, ^#^*P* < 0.05 (Student’s t-test).

Comparison of mitochondrial function between tissues requires a reliable normalization factor that corrects for non-mitochondrial protein contamination across subcellular isolations. For example, the activity of CS is commonly used as a marker of mitochondrial content for the normalization of a given bioenergetic readout. Although a variety of biochemical and protein biomarkers have been validated to reflect mitochondrial content in skeletal muscle^13^, the validity of these markers across other organs/tissues remains largely unexplored^12^. To determine potential biomarkers of mitochondrial content shared across organs, we isolated mitochondria from several distinct mouse tissues—brown adipose tissue (BAT), heart, kidney, and liver—and subjected them to label-free, quantitative nLC-MS/MS. The purity of each mitochondrial isolation (i.e., the percent contamination by non-mitochondrial proteins) was quantitatively evaluated by comparing the summed abundance of all mitochondrial proteins (i.e., MitoCarta 2.0 positive proteins) to total protein abundance. Note, total abundance reflects both mitochondrial and non-mitochondrial proteins. This allowed for the generation of a ‘mitochondrial enrichment factor’ (MEF) that reflects mitochondrial protein content per unit of crude isolated mitochondrial protein. Figure 1A depicts a heatmap of Log_2_ protein abundance from each mitochondrial sample (n = 5/tissue). In total, 2212 proteins were identified and quantified across all tissues, with 764 proteins distinguished as mitochondrial using the MitoCarta 2.0 database (Supplementary Table 1). Cluster analysis revealed that replicates from the same tissue type grouped together, demonstrating good reproducibility. Additionally, liver and kidney samples showed similar clustering to one another, as did BAT and heart samples. As expected, mitochondrial proteins accounted for a large percentage of all quantified proteins, yet the MEF varied considerably between tissues (Fig. 1B,C). Mitochondrial enrichment was highest in BAT and heart samples (> 90%), compared to kidney (~ 80%) and liver, with liver having the lowest purity (~ 65%) (Fig. 1C).

Using the same isolated mitochondria samples, we then compared CS activity across the four tissues to determine whether differences in CS activity matched those found in mitochondrial enrichment. The activity of CS was slightly higher in BAT compared to heart, with lower rates apparent in kidney and liver (Fig. 1D). A similar pattern was present with respect to CS abundance (Fig. 1E). Interestingly, the magnitude of difference in CS activity was much greater than that found for mitochondrial enrichment. This is particularly evident when comparing BAT and liver, for which there was an almost tenfold difference in CS activity (Fig. 1D), but < 1.5-fold difference in MEF (Fig. 1C). To directly determine whether CS activity predicted mitochondrial enrichment, linear regression was performed between CS activity rates and MEF for each tissue (Fig. 1F–I). Surprisingly, with the exception of liver, CS activity was not correlated with mitochondrial enrichment (Fig. 1F,I), indicating that CS activity is not a reliable marker of mitochondrial content across organs.

Having failed to validate CS activity as a biomarker of mitochondrial content, we turned our attention back to the proteome in an attempt to identify proteins whose expression correlated with mitochondrial enrichment across tissues. To do this, the expression of each identified/quantified mitochondrial protein was correlated with the sample-specific MEF (Supplementary Table 1). Although there were several proteins that correlated with mitochondrial enrichment among one or more tissues, no proteins were significantly correlated across all four tissues (Fig. 1J). Taken together, these data suggest that the activity and/or expression of a single protein is not a useful normalization factor for interpreting differences in mitochondrial physiology across tissues. Thus, throughout the rest of the manuscript, CS activity (Fig. 1K), as well as all other indices of bioenergetic flux were normalized to the sample-specific MEF (denoted with a * following the unit of measurement). For mitochondrial protein expression, nLC-MS/MS data were re-searched using the MitoCarta 2.0 database only. Using this approach, 795 mitochondrial proteins were identified and quantified across all four tissues (Supplementary Table 2). Pairwise comparisons of whole mitochondrial proteomes between tissues can be found in Supplementary Table 2. Importantly, we observed nearly identical proteome profiles using either the full mouse proteome database corrected for MitoCarta 2.0 abundance post-hoc or using the MitoCarta 2.0 database exclusively (Supplementary Fig. 1).

### Matrix metabolic enzymes display distinct expression and activity profiles across tissues

**Figure 2:**
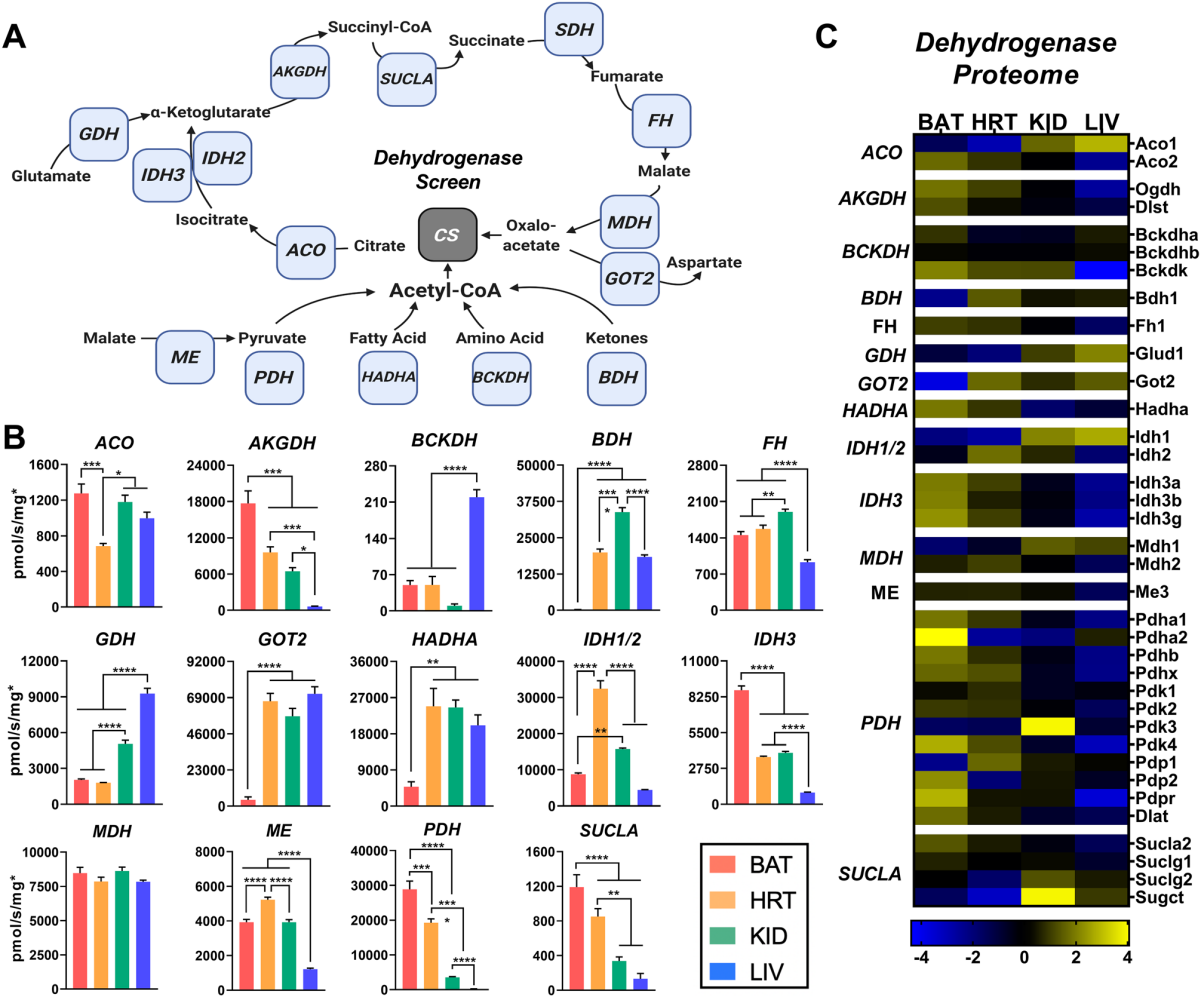
Intrinsic differences in matrix dehydrogenase activity and expression across tissues. (**A**) Cartoon depicting the matrix dehydrogenases, and associated enzymes, network. (**B**) Enzyme activity normalized to total protein and corrected for each sample’s MEF. (**C**) MitoCarta 2.0 normalized protein expression of individual enzyme subunits. (**A**) Figure created with BioRender.com. (**B,C**) Figure generated using GraphPad Prism 8 software (Version 8.4.2). Data information: Data are presented as mean ± SEM, N = 5/group. *P < 0.05, **P < 0.01, ***P < 0.001, ****P < 0.0001.

With the technical issue of normalization addressed, we sought to investigate inherent differences in mitochondrial function across tissues, starting with the mitochondrial dehydrogenase network. The dehydrogenase network consists of enzymes that comprise and feed into the citric acid cycle of the mitochondrial matrix (Fig. 2A). Therefore, we determined the activity (Fig. 2B) and expression (Fig. 2C) of matrix dehydrogenases in order to identify the carbon substrates that are most efficiently metabolized within each tissue. Figure 2C shows a heatmap of protein abundance for different subunits, isoforms, or regulatory proteins for the matrix enzymes. Each box represents the abundance of the indicated protein scaled to the tissue mean (see Supplementary Table 3 for statistical comparisons). In several cases, the protein expression data matched the enzymatic activity. For example, the activity and expression levels of both β-hydroxybutyrate dehydrogenase (BDH) and glutamic-oxaloacetic transaminase (GOT2) were extremely low in BAT. Interestingly, in the case of branched-chain α-ketoacid dehydrogenase (BCKDH), expression of the catalytic subunits of this enzyme complex were similar across all tissues, but expression of its kinase (BCKDK) was much lower in the liver (Fig. 2C, ‘BCKDH’). The absence of this regulatory kinase in the liver was reflected in significantly higher BCKDH activity (Fig. 2B, ‘BCKDH’). Another interesting observation was that the pattern of activity rates seen for pyruvate dehydrogenase (PDH; Fig. 2B, ‘PDH’) mirror those of CS activity (Fig. 1D), suggesting that CS activity could in fact be a reliable marker for PDH activity rather than mitochondrial content. This may explain the correlation between mitochondrial content and CS activity that others have observed in skeletal muscle^13^, as PDH is highly expressed in skeletal muscle and may happen to be proportionally expressed to mitochondrial number. Of all enzymes evaluated, only malate dehydrogenase (MDH) had equivalent activity rates across all four tissues (Fig. 2B, ‘MDH’). This may reflect the essential nature of MDH activity given its involvement in several metabolic pathways within the mitochondria.

### Heart mitochondria are the most metabolically flexible

**Figure 3:**
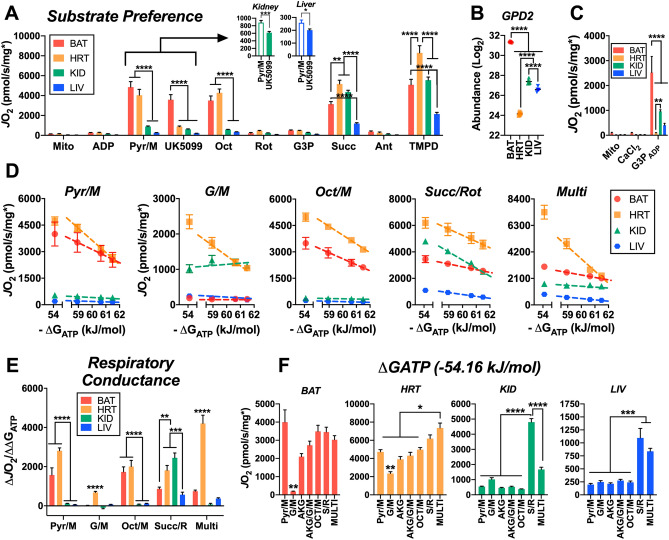
Carbon substrate preference and OXPHOS conductance across tissues. (**A**) Mitochondrial respiratory flux (*J*O_2_) stimulated with 0.5 mM ADP, clamped throughout the experiment via the hexokinase clamp. Substrate and inhibitor additions were as follows: Pyr/M (1 mM/1 mM), UK5099 (pyruvate carrier inhibitor – 0.001 mM), Oct (0.2 mM), Rot (0.0005 mM), G3P (10 mM), Succ (10 mM), Ant (0.0005 mM), TMPD (0.5 mM, plus 2 mM ascorbate). (**B**) Glycerol-3-phosphate dehydrogenase 2 (GPD2) abundance. (**C**) Mitochondrial *J*O_2_ in the presence of CaCl_2_ (1.5 µM), stimulated by G3P (10 mM) and 0.5 mM ADP via the hexokinase clamp (G3P_ADP_). (**D**) Relationship between *J*O_2_ and ATP free energy (ΔG_ATP_) clamped with the CK clamp in mitochondria energized with Pyr/M, G/M, Oct/M, Succ/Rot, and Multi. (**E**) Respiratory conductance – slope of the relationship between *J*O_2_ and ΔG_ATP_. (**F**) Maximal OXPHOS flux (*J*O_2_ at ΔG_ATP_ of − 54.16 kJ/mol) in response to various substrate combinations. Data information: (**A,D,F**) Data normalized to protein corrected for each sample’s MEF. (**C**) Data normalized to average MEF for that tissue. Figures generated using GraphPad Prism 8 software (Version 8.4.2) Data are Mean ± SEM, N = 5/group. *P < 0.05, **P < 0.01, ***P < 0.001, ****P < 0.0001.

We then set out to determine how differences in mitochondrial dehydrogenase activity between tissues may influence mitochondrial respiration when exposed to different energetic challenges and substrate conditions. We first probed the substrate preference of each set of tissues using a series of different carbon substrates and specific inhibitors (Fig. 3A). Throughout the assay, respiration was stimulated with saturating ADP using the hexokinase enzymatic clamp. The first observation was that heart mitochondria demonstrated a robust inhibition of pyruvate-mediated respiration following the addition of UK5099, an inhibitor of the common pyruvate carrier (Fig. 3A, ‘Pyr/M’ to ‘UK5099’). Although this effect was most obvious in the heart, Pyr/M supported respiration in BAT, as well as liver and kidney mitochondria was decreased by UK5099 (Fig. 3A, inset). Interestingly, relative to heart, percent inhibition by UK5099 in BAT mitochondria respiring on Pyr/M was lower. This may suggest that there is an alternate route for pyruvate transport into the mitochondrial matrix or an increased malate-alone supported rate of oxygen consumption (*J*O_2_) in BAT. In the presence of the fatty acid octanoyl-carnitine (Fig. 3A, ‘Oct’), further increases in *J*O_2_ were only observed in the heart, suggesting that heart mitochondria are better suited to oxidize fatty acids compared to the other tissues.

Following inhibition of NADH-linked flux with rotenone (Rot or R), glycerol-3-phosphate (Fig. 3A, ‘G3P’) did not induce an appreciable respiratory response in any of the tissues. This was puzzling, as previous work has shown that BAT typically displays high rates of G3P oxidation^14^. Furthermore, our proteomic data supported that the G3P dehydrogenase (GPD2) was highly expressed in BAT mitochondria (Fig. 3B). Previous work has shown that calcium is necessary for the activation of GPD2^15^, and thus the initial G3P oxidation rates observed herein may have been constrained by buffer EGTA. To control for this, respiration assays using G3P as a substrate were repeated in the presence of physiological free calcium (~ 1.5 µM). Inclusion of calcium in the assay buffer increased G3P-supported respiration in all tissues and normalized respiration rates aligned well with tissue-specific differences in GPD2 protein expression (Fig. 3B,C).

Only through the addition of the complex II substrate succinate (Succ or S) did the respiration rate of kidney approach that of the heart and surpass that of BAT. Although liver mitochondria also achieved a much greater rate with S as a substrate, the *J*O_2_ was comparatively lower with respect to BAT, heart, or kidney. This remained true when complex IV was stimulated directly using TMPD, a chemical that donates electrons directly to cytochrome C, thus circumventing the inhibition of complex III by antimycin A (Ant). Heart mitochondria had the greatest TMPD-stimulated *J*O_2_, while BAT and kidney were not different from one another (Fig. 3A, ‘TMPD’).

### Substrate preference differences across tissues are maintained across physiological ΔG_ATP_ spans

Next, we further explored the metabolic flexibility between tissues using a different energetic challenge: the more physiologically relevant creatine kinase (CK) clamp. This assay titrates ATP free energy (ΔG_ATP_) across a physiological span, from a high demand for ATP re-synthesis (ΔG_ATP_ = − 54.16 kJ) to a low demand state in which ATP free energy is highly charged (ΔG_ATP_ = − 61.49). If ADP phosphorylation is strongly coupled to respiration in a given tissue, then *J*O_2_ would be expected to be titrated down proportionally to the rise in ΔG_ATP_. Based upon the clear differences in fuel response we observed between tissues (Fig. 3A), we performed the CK clamp experiments under five distinct substrate conditions: Pyr/M; glutamate/M (G/M); Oct/M; Succ/Rot; α-ketoglutarate/G/M/Pyr/Oct/S (Multi). As seen in the substrate preference assay, the heart was able to achieve the highest maximal respiration rates across all substrate conditions (Fig. 3D, yellow squares). BAT mitochondria were the only tissue to match the *J*O_2_ of the heart, and this was observed only when fueled by Pyr/M (Fig. 3D, red circles). Paired with the results of the substrate preference assay, these data imply that across both the HK and CK clamp systems Pyr/M is the preferred respiratory substrate of BAT. Interestingly, we saw almost no flux in BAT mitochondria under the G/M condition. This observation is in line with our dehydrogenase activity and proteomic data which showed the lowest activity and expression of GOT2 in BAT (Fig. 2B,C, respectively). Respiration rates for liver and kidney were lowest across the entire ΔG_ATP_ titration under Pyr/M and Oct/M substrate conditions (Fig. 3D, green triangles and blue hexagons, respectively). Although this might imply a lower reliance on Complex I-supported respiration in both tissues, the kidney displayed slightly higher respiration when fueled by the Complex-I substrate G/M, approaching the rates of the heart (Fig. 3D, ‘G/M’). Most interestingly, the response to the CK clamp was altered in kidney mitochondria such that rather than consistently titrating respiration down as the ΔG_ATP_ charge was increased, respiration increased at the second titration point. This unexpected result may reflect either an ATP-dependent step in the oxidation pathway for G/M in kidney (i.e., ATP-mediated activation of metabolite oxidation), or possibly a slow activation of the enzymes involved in this pathway.

Beyond the absolute respiration rates themselves, the change in *J*O_2_ relative to the change in ΔG_ATP_ can be used to describe the sensitivity of mitochondria to changes in energy demand state. This is referred to as respiratory conductance and is calculated using the slope of the linear portion of the *J*O_2_/ΔG_ATP_ relationship (dashed lines in Fig. 3E). Conductance was highest in the heart across all four substrate conditions (Fig. 3E, yellow bars). BAT mitochondria matched the conductance of heart when fueled by NADH-linked substrates (excluding G/M), though conductance was halved under the Succ/Rot and Multi substrate conditions (Fig. 3E, red bars). The kidney also displayed an unusual substrate-dependent pattern to its conductance, in which Succ/Rot evoked the greatest values, but these were not maintained under the Multi substrate condition, in which Succ is present alongside multiple NADH-linked substrates (Fig. 3E, green bars). To determine how this might happen, we compared the maximal CK clamp-stimulated respiration rates across 7 substrate conditions (Fig. 3F), including α-ketoglutarate alone (AKG) and AKG/glutamate/M (AKG/G/M). Whereas combining all substrates for the Multi condition produced the greatest *J*O_2_ in heart mitochondria, respiration in kidney mitochondria was suppressed under the Multi condition compared to S/R alone (Fig. 3F).These data suggest that succinate is the predominant respiratory substrate of kidney mitochondria, and the presence of saturating amounts of NADH-linked substrates may restrict succinate uptake and/or oxidation by CII in the kidney.

### Respiratory complex expression differs by tissue

**Figure 4:**
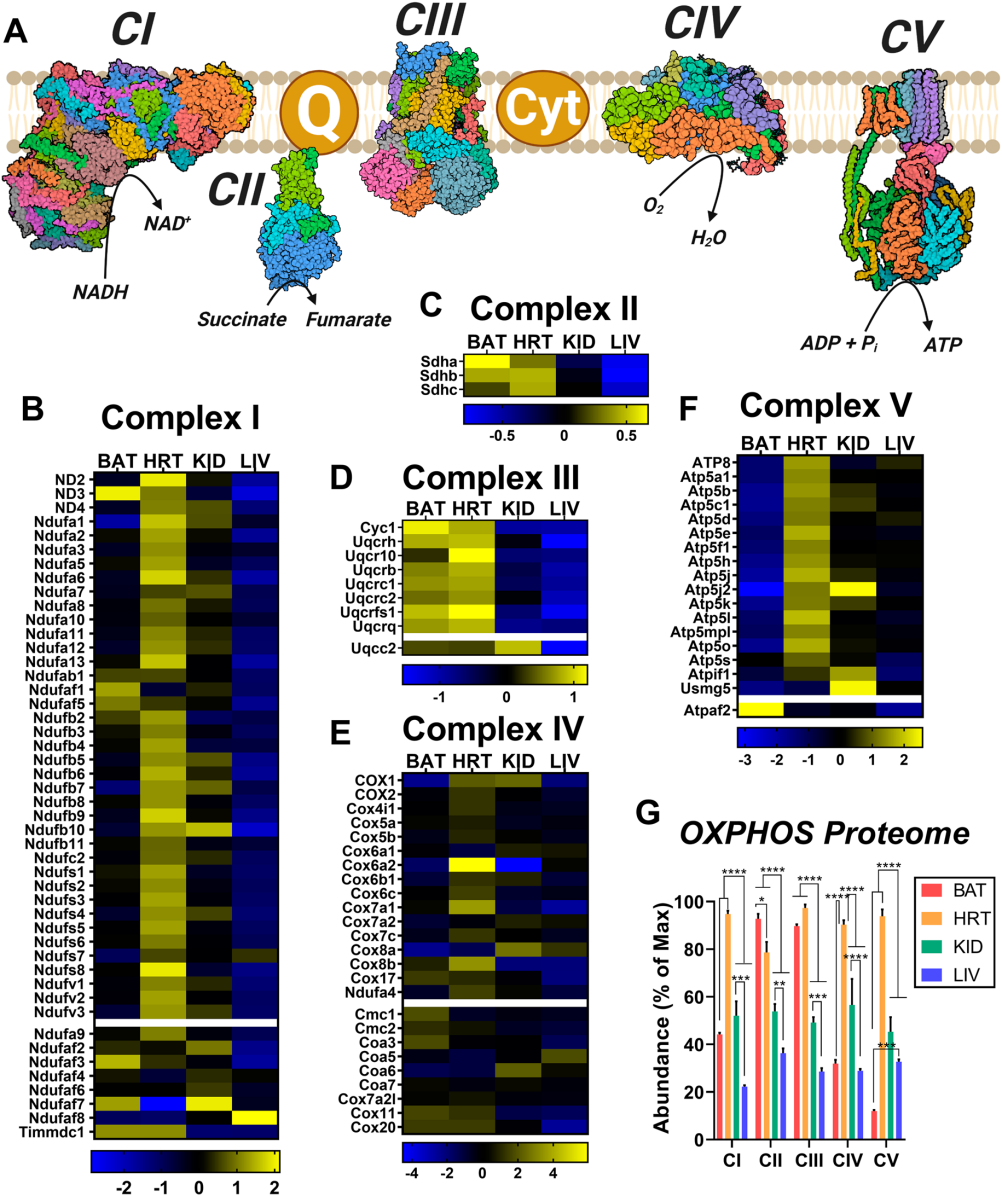
Intrinsic OXPHOS proteome heterogeneity across mouse tissues. (**A**) Cartoon depicting the protein complexes of the electron transport system and ATP synthase (i.e., CV). (**B–F**) MitoCarta 2.0 normalized protein expression of individual protein subunits of CI (**B**), CII (**C**), CIII (**D**), CIV (**E**), CV (**F**). (**G**) Quantification of the OXPHOS protein complexes generated by the summed abundance of all subunits within a given complex. Data are presented as a percentage of the max for each complex. (**A**) Figure created with BioRender.com. (**B–G**) Figure generated using GraphPad Prism 8 software (Version 8.4.2). Data information: Data are Mean ± SEM, N = 5/group. **P < 0.01, ***P < 0.001, ****P < 0.0001.

Given the vast differences in respiratory capacity and sensitivity, we then questioned whether there might also be intrinsic differences in the expression of the respiratory complexes across the four tissues. The mitochondrial OXPHOS system consists of five multi-enzyme complexes which collectively transfer electrons from various NAD- and FAD-linked dehydrogenases to O_2_ to form H_2_O. This stepwise transfer releases the energy necessary for complexes I, III, and IV to pump protons across the inner mitochondrial membrane, generating a proton gradient that is harnessed by complex V, ATP synthase, to phosphorylate ADP to ATP. The efficiency of this process depends upon the coordinated action of the various catalytic and accessory subunits that make up these complexes, as depicted in Fig. 4A. Therefore, changes in the expression of these subunits may have a significant impact upon respiration. To investigate, we searched our proteomics data for all proteins corresponding to components of the OXPHOS system and created separate expression heatmaps based on complex affiliation (Fig. 4B–F). As with the dehydrogenase protein expression heatmap (Fig. 2C), each box represents the mean abundance of the indicated protein in relation to the tissue mean (see Supplementary Table 3 for statistical comparisons). We also quantified relative complex expression between tissues by summing the abundance of all proteins related to a given complex and expressing each sum as a percentage of the max abundance across samples (Fig. 4G). Supporting the validity of our proteomic results, expression of Cox6a2, a subunit of complex IV specific to striated muscle^16^, was exclusively enriched in heart mitochondrial preparations (Fig. 4E). In general, the heart had the greatest expression of the majority of respiratory complexes, while the liver tended to have the lowest expression compared to the other tissues assessed (Fig. 4G). The only complex that was not highest in heart mitochondria was complex II, which is also the only complex that does not pump protons. As the tissue with the highest demand for ATP re-synthesis, the heart may not prioritize complex II expression as OXPHOS efficiency is highly dependent upon membrane potential polarization. Interestingly, overall complex I expression was found to be equivalent between BAT and kidney, despite vast differences in *J*O_2_ supported by complex I substrates Pyr/M and Oct/M (Fig. 4G). Further, the complex I expression of BAT was found to be approximately half that of heart despite similar Pyr/M-supported respiration rates. This may be reflective of a reserve of complex I that is present specifically in the heart due to its immense energetic demand. BAT also had the lowest expression of complex V of any tissue. The only protein from complex V that was highly expressed in BAT was Atpaf2, an assembly factor for the F1 component of ATP synthase (Fig. 4F).This, combined with its enrichment of Ucp1 (Supplementary Table 2), would suggest that OXPHOS is not the primary role of the electron transport system in BAT, entirely consistent with its known thermogenic function^17^.

### Maximal respiration rates differ by substrate and energetic challenge

**Figure 5:**
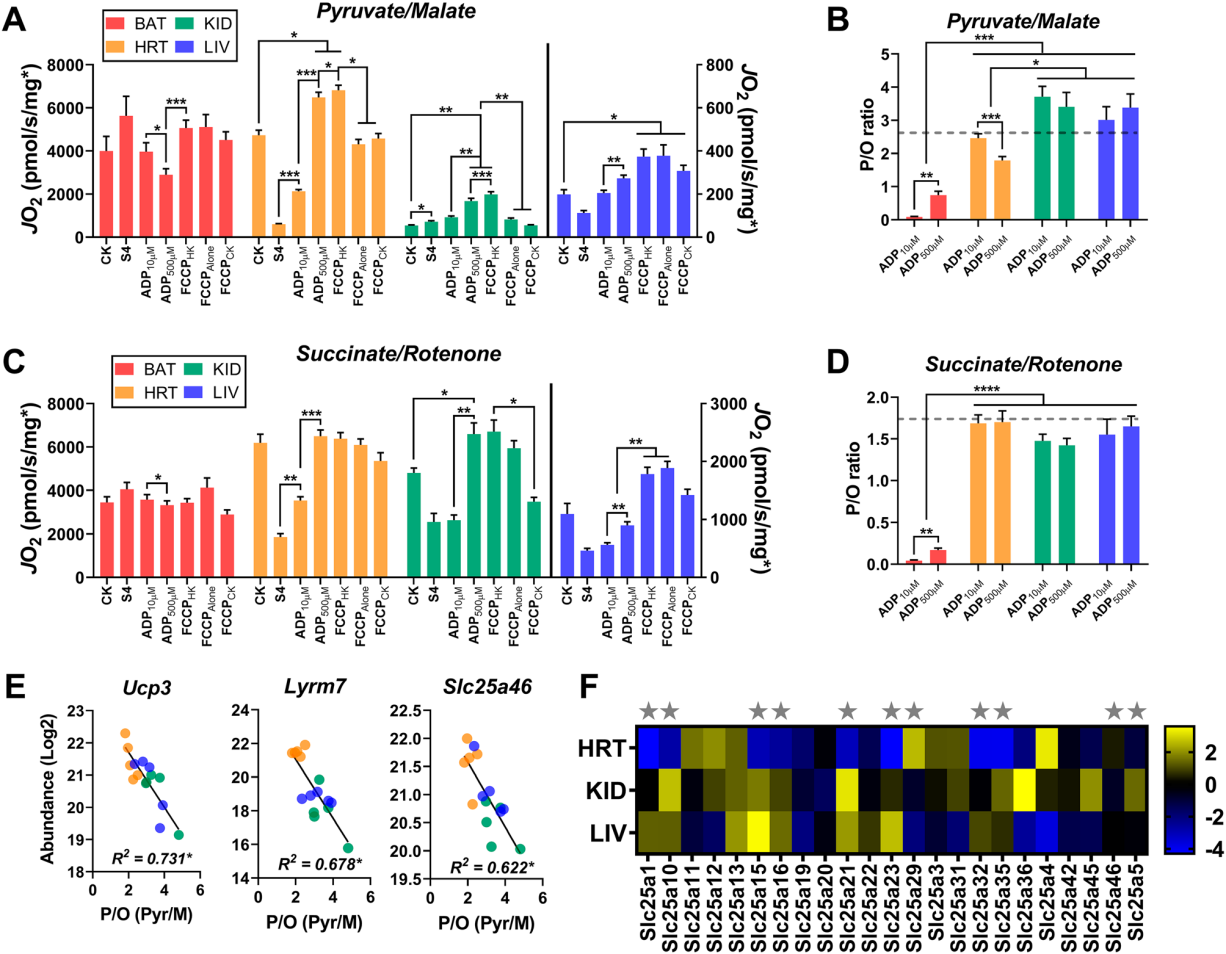
The mitochondrial SLC family of proteins correlate with Pyr/M supported intrinsic phosphorylation efficiency. (**A**) Respiration in isolated mitochondria energized with Pyr/M. Respiration was stimulated with ΔG_ATP_ of − 54.16 kJ/mol (CK), leak respiration in the absence of adenylates (S4), 0.01 mM ADP (ADP_10µM_), 0.5 mM ADP (ADP_500µM_), and FCCP in the presence of 0.5 mM ADP (FCCP_HK_), no adenylates (FCCP_Alone_), or ΔG_ATP_ (FCCP_CK_). (**B**) Relationship between mitochondrial ATP synthesis and oxygen consumption, a direct measurement of intrinsic phosphorylation efficiency (i.e., P/O ratio). Substrate condition = Pyr/M. Dashed line depicts the theoretical maximum for P/O based on the known mammalian stoichiometry of ATP synthesis. (**C**) Respiration in isolated mitochondria energized with succinate/rotenone. Respiration was stimulated with ΔG_ATP_ of − 54.16 kJ/mol (CK), leak respiration in the absence of adenylates (S4), 0.01 mM ADP (ADP_10µM_), 0.5 mM ADP (ADP_500µM_), and FCCP in the presence of 0.5 mM ADP (FCCP_HK_), no adenylates (FCCP_Alone_), or ΔG_ATP_ (FCCP_CK_). (**D**) Relationship between mitochondrial ATP synthesis and oxygen consumption, a direct measurement of intrinsic phosphorylation efficiency (i.e., P/O ratio). Substrate condition = succinate/rotenone. Dashed line depicts the theoretical maximum for P/O based on the known mammalian stoichiometry of ATP synthesis. (**E**) Correlation of MitoCarta 2.0 normalized protein abundance of Ucp3, Lyrm7, and Scl25a46 with Pyr/M supported P/O. (**F**) MitoCarta 2.0 normalized protein expression of identified/quantified mitochondrial SLC proteins. Stars indicate SLC proteins found to correlate with Pyr/M mediated P/O. Figures generated using GraphPad Prism 8 software (Version 8.4.2). Data information: Data are Mean ± SEM, N = 5/group. *P < 0.05, **P < 0.01, ***P < 0.001, ****P < 0.0001.

For a more complete picture of the unique responses to respiratory stimuli between tissues, we compiled the maximal respiration rates attained under several different simulated energetic challenges (Fig. 5A,B). To simplify, only two substrate conditions were selected—one primarily feeding complex I (Pyr/M; Fig. 5A), and the other feeding complex II (S/Rot; Fig. 5B). The state 4 (S4) rate represents *J*O_2_ stimulated by substrates in the absence of an adenylate challenge. In addition to the CK clamp (reproduced in Fig. 5A,B; ‘CK*’; ΔG_ATP_ = -54.16 kJ), maximal mitochondrial respiration was elicited through a two-point ADP titration using the HK clamp (‘ADP_10µM_’, ‘ADP_500µM_’) as well as the uncoupler FCCP in the absence of adenylates (‘FCCP_Alone_’) or following the completion of the CK and HK clamp experiments (‘FCCP_CK_’, ‘FCCP_HK_’, respectively).

Across both substrate conditions, the S4 respiration rate of BAT mitochondria was equivalent or greater than the rate attained under all energetic challenges (Fig. 5A,B, red bars). As this observation was specific to BAT, it is likely due to the expression of Ucp1, which was enriched in BAT mitochondria (Supplementary Table 2). Ucp1 allows reentry of protons from the intermembrane space into the mitochondrial matrix without passing through complex V, inflating the respiration rate through uncoupling^17^. Indeed, the S4 rate was equivalent to the maximal rate achieved by FCCP_Alone_ under both substrate conditions, suggesting that the mitochondria were uncoupled prior to the addition of adenylates (Fig. 5A,B). The presence of Ucp1 would also explain the stepwise decrease in respiration rate following the addition of increasing amounts of ADP (Fig. 5A,B), as ADP is a known inhibitor of Ucp1^18^.

Substrate condition had a notable influence over the response to different respiratory challenges in heart mitochondria. When fueled by S/Rot, all maximal energetic challenges (CK, ADP_500µM_, FCCP_Alone_, FCCP_CK_, and FCCP_HK_) evoked equivalent respiration rates (Fig. 5B, yellow bars). However, fueling with Pyr/M revealed a different pattern, in which the use of the HK clamp stimulated the greatest *J*O_2_ in the presence of saturating ADP and FCCP (Fig. 5A; ‘ADP_500µM_’, ‘FCCP_HK_’). This observation may reflect the different oxidation pathways of these two substrates, as oxidation of succinate by complex II directly feeds the electron transport system while multiple enzyme-mediated steps, including metabolite translocation across the inner mitochondrial membrane, are needed for the transfer of electrons from pyruvate and malate^19^. Given that the Pyr/M-supported FCCP_HK_ respiration rate was significantly greater than that of FCCP_Alone_ and FCCP_CK_ (Fig. 5A), it is also possible that a supraphysiological concentration of ADP may facilitate the activation of a greater number of respiratory complexes, or put a greater stress on the adenine nucleotide translocator (ANT), which has also been shown to directly transport protons^20^. As this effect is abolished under the S/Rot substrate condition (Fig. 5B), proton consumption by the metabolite translocation processes inherent to Pyr/M metabolism is the more likely culprit.

As seen earlier, mitochondria isolated from both the kidney and liver exhibited a clear preference for S/Rot-supported respiration over Pyr/M (Fig. 5A,B), likely due to their low PDH activity rates (Fig. 2B, PDH). Interestingly, we also observed an unusual bioenergetic phenotype in the kidney in which the S4 rate with Pyr/M, but not S/Rot, was greater than the maximal rate stimulated by the CK energetic clamp (Fig. 5A,B, green bars). This may be further evidence of the consumption of the proton gradient by the metabolite exchange processes linked to pyruvate oxidation. Indeed, as in the heart, the highest Pyr/M-supported respiration rates of kidney mitochondria were observed in the presence of the HK clamp and saturating ADP and FCCP (Fig. 5A, ‘ADP_500µM_’, ‘FCCP_HK_’). Regardless of substrate, liver mitochondria achieved their highest respiration rates when FCCP was the stimulus, not the adenylate-based CK or HK clamps, indicating that there are limitations to maximal electron transport in the mouse liver that are imposed by the OXPHOS system (Fig. 5A,B, blue bars).

### Efficiency of ATP production differs by substrate and tissue

We then expanded our interrogation of OXPHOS efficiency across the four tissues by directly quantifying the phosphorylation efficiency using the ratio of ATP production to *J*O_2_ (P/O ratio; Fig. 5C,D). ATP production was measured fluorometrically in a parallel HK clamp assay at both ADP concentrations. Both the respiration and fluorometric experiments were performed in the presence of Ap5A, an inhibitor of adenylate kinase (AK). Ap5A was included to eliminate non-OXPHOS-related production of ATP from two molecules of ADP by AK^21^. The dashed lines represent the theoretical maximum P/O ratios based upon the oxidation of NADH (P/O = 2.7; Fig. 5C) and succinate (P/O = 1.6; Fig. 5D). As would be expected given the high expression of Ucp1 (Supplementary Table 3) and low expression of CV previously noted (Fig. 4F), BAT demonstrated the lowest P/O ratios across all tissues under both substrate conditions, with ratios at both ADP concentrations far below the theoretical maximum (Fig. 5C,D, red bars).

Interestingly, in the heart, substrate condition again played a role in the efficiency of phosphorylation. Under the Pyr/M condition, the P/O ratio attained at ADP_10µM_ was already at the theoretical maximum, but then significantly dropped in response to an increased concentration of ADP (Fig. 5B, yellow bars), despite a large increase in respiration at ADP_500µM_ (Fig. 5A). This would suggest that the presence of a saturating amount of ADP may actually make the OXPHOS system less efficient, suggesting a trade-off between maximal respiratory flux and OXPHOS efficiency. The absence of this effect when the mitochondria are fueled by S/Rot (Fig. 5D) provides further evidence that there may be other processes involved in Pyr/M metabolism that may be consuming the proton gradient for non-OXPHOS purposes, which may include metabolite transport or proton leak.

Surprisingly, despite their limited respiration under the Pyr/M substrate condition, both kidney and liver appeared to be generating appreciable amounts of ATP (Fig. 5B, green and blue lines, respectively). In fact, their Pyr/M-fueled P/O values were greater than those of the heart, as well as the theoretical maximum P/O ratio. As the theoretical maximum was calculated based upon the stoichiometry of the c-subunit composition of the mammalian ATP synthase and the number of protons required for one full turn of the central stalk^22^, this observation has two possible explanations. One would be that the stoichiometry of the ATP synthase is not fixed, which would imply that there is not a single theoretical maximum. This does not seem likely as the heart, kidney, and liver all displayed similar P/O ratios that were equivalent to the theoretical maximum when supported by S/Rot (Fig. 5D). Alternatively, there may have been a contribution of ATP generated through substrate-level phosphorylation rather than OXPHOS. One potential site of substrate-level phosphorylation could be succinyl-CoA synthetase, which is known to have the ability to generate ATP rather than GTP, and this enzyme would not be active in the S/Rot substrate condition. However, this conclusion is not supported by our protein expression data from Fig. 2C, in which the subunit that favors ADP as a substrate (Sucla2) is shown to be relatively depleted in kidney and liver compared to BAT and heart. Another possibility is that these mitochondrial populations express a version of AK that is insensitive to Ap5A that was responsible for the elevated ATP production. Lastly, given than the *J*ATP synthesis and *J*O_2_ assays were run in parallel, rather than being quantified simultaneously^23^, such conditions may have resulted in slight overestimation of P/O.

### Protein predictors of intrinsic OXPHOS efficiency across tissues are enriched for mitochondrial transporters

Given the increased variability in Pyr/M-supported P/O ratios, we sought to determine whether there were any proteins that were predictive of OXPHOS efficiency across tissues. We searched our proteomic data for the mitochondrial proteins that were most highly correlated with Pyr/M-supported P/O ratio in heart, kidney, and liver tissues. BAT was excluded from this analysis due to the confounding variable of Ucp1 expression. The top 3 most highly correlated proteins are plotted in Fig. 5E. Ucp3 expression was found to be negatively correlated with the efficiency of OXPHOS (Fig. 5E, Ucp3), which would be expected given its role as a mitochondrial uncoupling protein^24,25^. Lyrm7 expression was also found to be negatively correlated to P/O (Fig. 5E, Lyrm7). It functions as an assembly factor that chaperones the insertion of the Rieske iron-sulfur protein Uqcrfs1 into complex III, a role that may lead to its elevated expression during mitochondrial repair^26^. Finally, Slc25A46 belongs to a family of solute carrier proteins, many of which use the mitochondrial proton gradient to transport metabolites in and out of the mitochondria. Given that many of the SLC family of proteins dissipate the proton motive force to do non-OXPHOS work, it is intuitive that they would be negatively correlated with phosphorylation efficiency (Fig. 5E, Slc25A46). In fact, as we probed further into the proteomics data for other members of the SLC family, we noted that almost half of the mitochondrial SLC proteins that were identified were significantly correlated with the P/O ratio (Fig. 5F; stars denote a significant correlation).

## Discussion

Citrate synthase activity has commonly been the standard strategy for normalization between mitochondrial samples, though, to our knowledge, this has not been validated for comparisons across different tissues. To address this issue, we determined the mitochondrial purity of each sample by calculating the proportion of all nLC-MS/MS-quantified proteins that could be identified as mitochondrial using the MitoCarta 2.0 database. This provided a mitochondrial enrichment factor (MEF) for each sample that could be compared across tissues. Our findings indicate that CS activity was not significantly correlated with the MEF for three out of the four tissues studied. In fact, there were no identified mitochondrial proteins that universally correlated with the MEF. As no protein surrogate was found to be an adequate estimate of mitochondrial purity, the MEF is potentially the optimal method for normalization across tissue types when performing mitochondrial isolations. Although previous work has utilized a similar mass spectrometry-based normalization method in mouse liver^27^, this may be the first true comparison of intrinsic mitochondrial respiratory kinetics between several different mammalian tissues.

Collectively, our findings concerning the bioenergetic phenotype of BAT agree with those previously published in the literature^28^. Respiration was uniformly high across all substrate conditions (with the exception of glutamate), even in the absence of an adenylate-based stimulus (i.e. state 4 respiration), likely due to the expression of Ucp1 in BAT mitochondria. Interestingly, Ucp1 was only one limitation to the maximal phosphorylation efficiency of BAT, as it was also shown that expression of complex V, or ATP synthase, was significantly depressed in BAT compared to all other tissues. Another curious observation was that expression of complex I-related proteins in BAT was nearly half that of the heart, yet respiration rates for most complex I substrates (including pyruvate, AKG, and octanoyl-carnitine) were generally comparable between BAT and heart. This finding may imply that in the presence of an uncoupling protein, which allows more free cycling of protons across the inner mitochondrial membrane compared to the demand-driven complex V, a smaller number of respiratory complexes may be necessary to achieve the same rate of electron flow.

Mitochondria isolated from the heart, the tissue with the highest energetic demand of the four studied, predictably had the highest maximal respiration rates under nearly every condition. Heart mitochondria were also the only to display an additive effect to respiration when energized by the Multi substrate condition, highlighting the metabolic flexibility of their mitochondrial network. As might be expected, the conductance of the heart, an estimate of bioenergetic efficiency in response to changing ΔG_ATP_, was among the highest under every substrate condition. When phosphorylation efficiency was quantified directly, the heart was able to attain the theoretical maximum P/O values at the lowest concentration of ADP when energized by both Pyr/M and S/Rot. Interestingly, however, the presence of saturating ADP was shown to decrease the P/O ratio below the theoretical maximum when energized by Pyr/M, but not S/Rot. Given that there was a large increase in respiration with the greater concentration of ADP, the decrease in P/O would suggest that this jump in *J*O_2_ was not met with a proportional increase in ATP production. One possibility for this discrepancy is that supra-physiological concentrations of ADP may activate a ‘reserve capacity’ of additional respiratory complexes in order to meet this greater demand, albeit with a lower phosphorylation efficiency. This explanation would align with the discrepancy in complex expression between BAT and heart. Alternatively, such a high ADP concentration may also stress ANT, which is responsible for exchanging ADP and ATP at the inner mitochondrial membrane. This transport process is powered by the proton gradient in the same way as complex V, and ANT has additionally been shown to transport protons into the matrix independently of its ADP/ATP transport activity^20^. Proton movement through ANT would thus mimic the effects of an uncoupling protein.

As an organ whose primary responsibility is to maintain concentration gradients across membranes, the kidney would be expected to have a high demand for ATP re-synthesis. Our results suggest that succinate is the preferred respiratory substrate for the kidney, across a variety of stimulus conditions, consistent with previous findings^29^. However, the kidney was the only tissue to demonstrate significantly depressed respiration under a Multi substrate condition. This may have important implications in the case of diet-induced kidney disease, as it is apparent that their mitochondria may be functionally impaired in the presence of excess NAD-linked carbon substrates^30^. More interestingly, the kidney was the only tissue to demonstrate an increase in respiration in response to the CK clamp-imposed titration of ΔG_ATP_. This effect was only seen when the kidney was fueled by glutamate/malate, which may reflect an ATP-dependent step in the glutamate oxidation pathway. This would not be entirely unusual as a similar phenomenon was recently demonstrated with respect to the oxidation of branched-chain keto-acids^31^.

Although the kidney and liver appear to favor succinate over complex I substrates to support respiration, both displayed P/O values that were greater than the theoretical maximum values when supported by Pyr/M. The theoretical maximum values are based on the stoichiometry of the number of protons that it would take to fully turn the rotor of complex V, which generates three ATP molecules^22^. This discrepancy may be caused by several different scenarios. First, it is possible that this stoichiometry is not fixed, as has been previously thought. This is unlikely, given that the succinate-supported P/O remained at or below the theoretical maximum for both tissues. Second, a portion of the ATP production that was observed could be through substrate-level phosphorylation. The most likely candidate for this would be SUCLA, which can express an isoform that prefers ADP rather than GDP as its substrate. This possibility is supported by the fact that P/O does not remain above the maximum when fueled by succinate, as SUCLA would no longer be involved in substrate catalysis. From our proteomic data, it appears that liver and kidney preferentially express the GDP-favoring Suclg2 isoform. However, it is also possible that these tissues express an enzyme which catalyzes the interconversion from GTP to ATP. Finally, this measurement was obtained using parallel experiments in a respiration chamber and a fluorometer, which may be a technical limitation to obtaining accurate P/O values. Regardless, the pattern that was observed in which the Pyr/M-supported P/O values obtained for liver and kidney were greater than those of heart is intriguing.

One of the most fascinating findings was the high proportion of SLC family proteins^32,33^ whose expression were significantly correlated to phosphorylation efficiency. This relationship makes sense as many of the SLC proteins as well as complex V consume the proton gradient to perform cellular work. Thus, if both SLC proteins and complex V are active at the same time, some proportion of the *J*O_2_ must be used to power the SLC rather than generate ATP, lowering the P/O.

As studies of bioenergetic function advance, it is becoming more apparent that not all mitochondria are created equal. Full appreciation of the metabolic specialization of different tissues with different energetic demands can only be achieved through normalization that factors in the purity of the mitochondrial sample. Our results support the use of mitochondrial-targeted nLC-MS/MS to determine mitochondrial enrichment on a per-sample basis. This method will allow for unbiased comparison of functional parameters between populations of mitochondria isolated from metabolically distinct tissues, as well as in response to a variety of physiological (e.g., exercise, fasting) and pathophysiological (e.g., diabetes, cancer) stressors.

## Materials and methods

### Animals

Animal experiments were performed using C3H/HeJ mice (n = 10; The Jackson Laboratory, stock #000,659) according to the relevant guidelines approved by the East Carolina University Institutional Animal Care and Use Committee. At the time of tissue harvest, 12 h-fasted mice were anesthetized with isofluorane, and brown adipose (subscapular; BAT), heart, kidney, and liver tissues were removed and immediately subjected to mitochondrial isolation.

### Chemicals and reagents

Unless otherwise stated, all chemicals were purchased from Sigma-Aldrich.

### Mitochondrial isolation

Mitochondria were isolated from all tissues using differential centrifugation as described previously, with some modifications^34^. The following buffers were utilized: Buffer A—MOPS (50 mM), KCl (100 mM), EGTA (1 mM), MgSO_4_ (5 mM), pH = 7.1; Buffer B—Buffer A, supplemented with bovine serum albumin (BSA; 2 g/L). After removal, all tissues were immediately placed in ice-cold Buffer B, minced, then homogenized via a drill-driven Teflon pestle and borosilicate glass vessel. Homogenates were centrifuged at 800×*g* for 10 min at 4 °C. The supernatant was filtered through gauze and centrifuged at 10,000×*g* for 10 min at 4 °C. The pellets were washed in 1.4 mL of Buffer A, transferred to microcentrifuge tubes, and again centrifuged at 10,000×*g* for 10 min at 4 °C. Final mitochondrial pellets were resuspended in 100–200µL of Buffer A. Protein content was determined via the Pierce BCA protein assay. Freshly prepared mitochondria were then used for citrate synthase activity and functional experiments, with a portion of each mitochondrial suspension flash frozen for matrix dehydrogenase activity and proteomic analyses. Using this method, a single mitochondrial preparation can be used to generate all biochemical outcomes described in this paper.

### Citrate synthase activity

Citrate synthase (CS) activity was determined using a colorimetric plate-based assay in which CoA-SH, a byproduct formed by the CS-mediated reaction of oxaloacetate and acetyl-CoA, interacts with 5′, 5′-Dithiobis 2-nitrobenzoic acid (DTNB) to form TNB (OD: 412 nm). Assay buffer consisted of Buffer C (105 mM potassium-MES, 30 mM KCl, 10 mM KH_2_PO_4_, 5 mM MgCl_2_, and 1 mM EGTA; pH = 7.2) supplemented with DTNB (0.2 mM) and acetyl-CoA (0.5 mM). A 96-well round bottom plate was loaded with assay buffer (200 µL/well), the permeabilizing agent alamethicin (0.03 mg/mL), and isolated mitochondria (10 µg/well) and then incubated at 37 °C for 5 min to deplete endogenous substrates. The assay was initiated by the addition of oxaloacetate (1 mM) to sample wells, with absorbance at 412 nm recorded every 30 s for 20 min. The mitochondrial suspension was also added to one control well per sample to account for nonspecific activity, which was later subtracted from the sample rate. CS activity was determined using the Beer-Lambert Law and the molar absorption coefficient of TNB (13.6 mM/cm).

### Mitochondrial functional assessment

High-resolution O_2_ consumption rate (*J*O_2_) measurements were conducted using the Oroboros Oxygraph-2 K (Oroboros Instruments, Innsbruck, Austria). The base assay buffer was Buffer C (105 mM potassium-MES, 30 mM KCl, 10 mM KH_2_PO_4_, 5 mM MgCl_2_, 1 mM EGTA, 2.5 g/L BSA and 5 mM creatine monohydrate; pH = 7.2), modified to simulate 3 different energetic challenges: the hexokinase enzymatic clamp (HK clamp); the creatine kinase energetic clamp (CK clamp); and titration of uncoupling agent carbonyl cyanide-*4*-(trifluoromethoxy)phenylhydrazone (FCCP). The HK clamp maintained a steady-state ADP concentration by consuming any newly generated ATP to phosphorylate glucose^21^. The CK clamp titrated the free energy of ATP hydrolysis (ΔG_ATP_) as described below. Titration of FCCP was used to stimulate *J*O_2_ independent of the demand for ATP re-synthesis. All respiration experiments were carried out at 37 °C in a 1 mL reaction volume, with 25-300 µg protein loaded per experiment.

#### Substrate preference assay

Substrate preference was assessed by comparing the steady-state *J*O_2_ attained following sequential additions of different carbon substrates and specific inhibitors in the presence of a maximal ADP concentration (500 µM) maintained using the HK clamp. Assay buffer was supplemented with HK (1 U/ml) and glucose (5 mM). Assay was initiated through addition of mitochondria, followed by ADP and pyruvate/malate (P/M; 1 mM/2 mM). The pyruvate carrier inhibitor acyano-(1-phenylindol-3-yl)-acrylate (UK5099; 1 µM) was then added to prevent pyruvate entry, followed by octanoyl-carnitine (Oct; 0.2 mM). NADH-linked respiration was then inhibited using rotenone (Rot; 0.05 µM), and glycerol-3-phosphate (G3P; 10 mM) was added. G3P feeds electrons into the ubiquinone pool through the mitochondrial G3P dehydrogenase located on the outer portion of the inner mitochondrial membrane. Succinate (S; 10 mM) was then added to stimulate complex II-linked respiration. Complex III was then inhibited by antimycin A (Ant; 0.005 µM), and complex IV was stimulated through the addition of the cytochrome C-specific electron donor N,N,N’,N’-tetramethyl-*p*-phenylenediamine (TMPD; 0.5 mM dissolved in ascorbate – final ascorbate concentration of 2 mM).

In a separate experiment using a subset of five C3H mice, we determined G3P-supported respiration in the presence of calcium, which is necessary to fully activate the G3P dehydrogenase^15^. For this experiment, mitochondria were added to Buffer C, followed by an addition of 0.8 mM of CaCl_2_ (1.5 µM free calcium). G3P (10 mM) was then added, followed by the HK clamp components and ADP (500 µM).

#### Creatine kinase clamp experiments

As previously described, a modified version of the CK clamp technique was used to determine steady-state *J*O_2_ across a physiological span of ΔG_ATP_ using known amounts of creatine (Cr), phosphocreatine (PCr), and ATP in the presence of excess amounts of CK ^19,35,36^. For complete details regarding the calculation of ΔG_ATP_ at each titration point see^19^. To begin, mitochondria were added to Buffer C, followed by the addition of respiratory substrates to stimulate State 4 respiration. The CK clamp was then initiated by the addition of ATP (5 mM), PCr (1 mM), and CK (20U/ml), simulating a ‘maximal’ demand for ATP re-synthesis. Sequential additions of PCr to 6, 15, and 21 mM were then performed to gradually lower the ATP demand state back toward baseline. After the final PCr addition, FCCP was titrated (0.5, 1, 2 µM) to stimulate respiration back up towards maximal *J*O_2_. Plotting the calculated ΔG_ATP_ against the corresponding steady-state *J*O_2_ reveals a linear force-flow relationship, the slope of which represents the conductance/sensitivity of the entire respiratory system under the specified substrate constraints. Five substrate conditions were used: Pyr/M (5 mM/2 mM); glutamate/M (G/M; 10 mM/2 mM); Oct/M (0.2 mM/2 mM); S/Rot (10 mM/0.5 µM); α-ketoglutarate/G/M/Pyr/Oct/S (abbreviated as Multi; 10 mM/5 mM/2 mM/5 mM/0.2 mM/5 mM).

#### ATP/O assay

Parallel respiration and fluorometric ADP titration experiments were carried out in order to generate an ATP/O ratio. Fluorometry experiments were carried out using a QuantaMaster Spectrofluorometer (QM-400; Horiba Scientific) at 37 °C in a 200µL reaction volume with continuous stirring. All experiments were conducted using the HK clamp to maintain the desired ADP concentration. Assay buffer was supplemented with HK (1U/mL), glucose (5 mM), glucose-6-phosphate dehydrogenase (2U/mL), and NADP^+^ (4 mM), as well as P1,P5-di(adenosine-5′)pentaphosphate (Ap5A; 0.2 µM). Ap5A was included to inhibit adenylate kinase, preventing the generation of ATP from ADP alone^21^. To begin, mitochondria were added, followed by respiratory substrates. ADP was titrated to 10 and 500 µM. Respiration experiments were then continued with an FCCP titration (0.5, 1, 2 µM). Fluorometry experiments were conducted in both the presence and absence of oligomycin (0.02 µM) to account for fluorescence unrelated to ATP synthase activity. The rate of change in NAD(P)H fluorescence (Ex:Em, 350:450) was equated to the rate of ATP production (*J*ATP), as previously described^21^. The ATP/O ratio was then calculated as *J*ATP/*J*O_2_, divided by 2. Respiratory substrates were limited to complex I-specific Pyr/M (5 mM/2 mM) or complex II-specific S/Rot (10 mM/0.5 µM).

### Matrix dehydrogenase activity assays

Enzymatic activities of matrix dehydrogenases were determined via the autofluorescence of NADH or NADPH (Ex:Em 340:450), as described previously^19^. Evaluated enzymes included aconitase (ACO), α-ketoglutarate dehydrogenase (AKGDH), branched-chain α-ketoacid dehydrogenase (BCKDH), β-hydroxybutyrate dehydrogenase (BDH), fumarate hydratase (FH), glutamate dehydrogenase (GDH), glutamic-oxaloacetic transaminase (GOT2), β-hydroxyacyl-CoA dehydrogenase (HADHA), isocitrate dehydrogenase 2 (IDH2), isocitrate dehydrogenase 3 (IDH3), malate dehydrogenase (MDH), malic enzyme (ME), pyruvate dehydrogenase (PDH), and succinyl-CoA synthetase (SUCLA). With the exception of SUCLA, all activity assays were performed in a 96-well plate in a 200µL volume. SUCLA activity was determined using the same assay volume in the QM-400 fluorometer. Fluorescence was measured every minute for 60 min in the plate reader and continuously in the fluorometer. Enzymatic rates were determined using a standard curve performed with NADH standards ranging from 20 to 50,000 pM.

### Mitochondrial lysis and sample prep for label-free proteomics

Isolated mitochondria were lysed in Buffer D (8 M urea in 40 mM Tris, 30 mM NaCl, 1 mM CaCl_2_, 1 × cOmplete ULTRA mini EDTA-free protease inhibitor tablet; pH = 8.0), as described previously^37^. The samples were subjected to three freeze–thaw cycles, and sonication with a probe sonicator in three 5 s bursts (Q Sonica #CL-188; amplitude of 30). Samples were then centrifuged at 10,000 × g for 10 min at 4 °C. Protein concentration was determined by BCA protein assay. Equal amounts of protein were reduced with 5 mM DTT at 37 °C for 30 min, and then alkylated with 15 mM iodoacetamide at room temperature for 30 min in the dark. Unreacted iodoacetamide was quenched with DTT up to 15 mM. Initial digestion was performed with Lys C (ThermoFisher Cat# 90,307; 1:100 w:w; 2 µg enzyme per 200 µg protein) for 4 h at 37 °C. Following dilution to 1.5 M urea with 40 mM Tris (pH = 8.0), 30 mM NaCl, 1 mM CaCl_2_, samples were digested overnight with trypsin (Promega; Cat# V5113; 50:1 w/w, protein:enzyme) at 37 °C. Samples were acidified to 0.5% TFA and then centrifuged at 4000×*g* for 10 min at 4 °C. Supernatant containing soluble peptides was desalted, as described previously^37^ and then eluate was frozen and lyophilized.

### nLC-MS/MS for label-free proteomics

Final peptides were resuspended in 0.1% formic acid, quantified (ThermoFisher Cat# 23,275), and then diluted to a final concentration of 0.25 µg/µL. Samples were subjected to nLC-MS/MS analysis using an UltiMate 3000 RSLCnano system (ThermoFisher) coupled to a Q Exactive Plus Hybrid Quadrupole-Orbitrap mass spectrometer (ThermoFisher) via a nanoelectrospray ionization source. For each injection, 4µL (1 µg) of sample was first trapped on an Acclaim PepMap 100 20 mm × 0.075 mm trapping column (ThermoFisher Cat# 164,535; 5 μL/min at 98/2 v/v water/acetonitrile with 0.1% formic acid). Analytical separation was then performed over a 95 min gradient (flow rate of 250nL/min) of 4–25% acetonitrile using a 2 µm EASY-Spray PepMap RSLC C18 75 µm × 250 mm column (ThermoFisher Cat# ES802A) with a column temperature of 45ºC. MS1 was performed at 70,000 resolution, with an AGC target of 3 × 10^6^ ions and a maximum injection time (IT) of 100 ms. MS2 spectra were collected by data-dependent acquisition (DDA) of the top 15 most abundant precursor ions with a charge greater than 1 per MS1 scan, with dynamic exclusion enabled for 20 s. Precursor ions isolation window was 1.5 m/z and normalized collision energy was 27. MS2 scans were performed at 17,500 resolution, maximum IT of 50 ms, and AGC target of 1 × 10^5^ ions.

### Data analysis for label-free proteomics

As described previously^37^, with some modification, Proteome Discoverer 2.2 (PDv2.2) was used for raw data analysis, with default search parameters including oxidation (15.995 Da on M) as a variable modification and carbamidomethyl (57.021 Da on C) as a fixed modification. Data were searched against the Uniprot Mus musculus reference proteome (Proteome ID: UP 000000589), as well as the mouse Mito Carta 2.0 database^38^. PSMs were filtered to a 1% FDR and grouped to unique peptides while maintaining a 1% FDR at the peptide level. Peptides were grouped to proteins using the rules of strict parsimony and proteins were filtered to 1% FDR. Peptide quantification was done using the MS1 precursor intensity. Imputation was performed via low abundance resampling. Using only high confidence master proteins, mitochondrial enrichment factor (MEF) was determined by comparing mitochondrial protein abundance (i.e., proteins identified to be mitochondrial by cross-reference with the MitoCarta 2.0 database) to total protein abundance.

### Statistical evaluation

All mass spectrometry samples were normalized to total protein abundance, and the protein tab in the PDv2.2 results was exported as a tab delimited .txt. file and analyzed. Protein abundance was converted to the Log_2_ space. For pairwise comparisons, tissue mean, standard deviation, p-value (p; two-tailed Student’s t-test, assuming equal variance), and adjusted p-value (Benjamini Hochberg FDR correction) were calculated^39,40^. Multi-tissue comparisons were analyzed by 2-way ANOVA (Tissue x Protein) using Tukey’s test to correct for multiple comparisons. Where indicated, Pearson correlation coefficients were generated using GraphPad Prism 8 software (Version 8.4.2).

Functional assay results are expressed as the mean ± SEM (error bars). Data were normalized to protein loaded per experiment and then corrected for the mitochondrial enrichment factor (*) calculated for that sample, with the final values expressed as pmol/s/mg protein*. Differences between tissues were assessed by one-way ANOVA, followed by Tukey’s test where appropriate using GraphPad Prism 8 software (Version 8.4.2). Other statistical tests used are described in the figure legends. Statistical significance in the figures is indicated as follows: *p < 0.05; **p < 0.01; ***p < 0.001; ****p < 0.0001. Unless otherwise stated, figures were generated using GraphPad Prism 8 software (Version 8.4.2).

## Supplementary information

Supplementary Information

Supplementary Table 1

Supplementary Table 2

Supplementary Table 3

Supplementary Table 4

## Data Availability

All raw data for proteomics experiments are available online using accession number “JPST000908” for jPOST Repository^41,42^. All bioenergetic flux data for each sample are provided in Supplementary Table 4.

## References

[1] Gaude E, Frezza C (2014). Defects in mitochondrial metabolism and cancer. Cancer Metab..

[2] Fisher-Wellman KH, Neufer PD (2012). Linking mitochondrial bioenergetics to insulin resistance via redox biology. Trends Endocrinol. Metab..

[3] Che R, Yuan Y, Huang S, Zhang A (2014). Mitochondrial dysfunction in the pathophysiology of renal diseases. Am. J. Physiol. Renal Physiol..

[4] Diaz-Vegas A (2020). Is Mitochondrial dysfunction a common root of noncommunicable chronic diseases?. Endocr. Rev..

[5] Frezza C, Cipolat S, Scorrano L (2007). Organelle isolation: functional mitochondria from mouse liver, muscle and cultured filroblasts. Nat. Protoc..

[6] van der Walt G, Louw R (2020). Novel mitochondrial and cytosolic purification pipeline for compartment-specific metabolomics in mammalian disease model tissues. Metabolomics.

[7] Glancy B, Balaban RS (2011). Protein composition and function of red and white skeletal muscle mitochondria. Am. J. Physiol. Cell Physiol..

[8] Kappler L (2016). Purity matters: a workflow for the valid high-resolution lipid profiling of mitochondria from cell culture samples. Sci. Rep..

[9] Thor Johnson D (2007). Tissue heterogeneity of the mammalian mitochondrial proteome. Am. J. Physiol. Cell Physiol..

[10] Johnson DT, Harris RA, Blair PV, Balaban RS (2007). Functional consequences of mitochondrial proteome heterogeneity. Am. J. Physiol. Cell Physiol..

[11] Forner F, Foster LJ, Campanaro S, Valle G, Mann M (2006). Quantitative proteomic comparison of rat mitochondria from muscle, heart, and liver. Mol. Cell. Proteomics.

[12] Groennebaek T (2020). Utilization of biomarkers as predictors of skeletal muscle mitochondrial content after physiological intervention and in clinical settings. Am. J. Physiol. Endocrinol. Metab..

[13] Larsen S (2012). Biomarkers of mitochondrial content in skeletal muscle of healthy young human subjects. J. Physiol..

[14] Mráček T, Drahota Z, Houštěk J (2013). The function and the role of the mitochondrial glycerol-3-phosphate dehydrogenase in mammalian tissues. Biochim. Biophys. Acta..

[15] Clarke KJ, Porter RK (2018). The importance of calcium ions for determining mitochondrial glycerol-3-phosphate dehydrogenase activity when measuring uncoupling protein 1 (UCP1) function in mitochondria isolated from brown adipose tissue. Methods Mol. Biol..

[16] Inoue M (2019). COX6A2 variants cause a muscle-specific cytochrome c oxidase deficiency. Ann. Neurol..

[17] Harms M, Seale P (2013). Brown and beige fat: development, function and therapeutic potential. Nat. Med..

[18] Divakaruni AS, Humphrey DM, Brand MD (2012). Fatty acids change the conformation of uncoupling protein 1 (UCP1). J. Biol. Chem..

[19] Fisher-Wellman KH (2018). Mitochondrial diagnostics: a multiplexed assay platform for comprehensive assessment of mitochondrial energy fluxes. Cell Rep..

[20] Bertholet AM (2019). H+ transport is an integral function of the mitochondrial ADP/ATP carrier. Nature.

[21] Lark DS (2016). Direct real-time quantification of mitochondrial oxidative phosphorylation efficiency in permeabilized skeletal muscle myofibers. Am. J. Physiol. - Cell Physiol..

[22] Watt IN, Montgomery MG, Runswick MJ, Leslie AGW, Walker JE (2010). Bioenergetic cost of making an adenosine triphosphate molecule in animal mitochondria. Proc. Natl. Acad. Sci. U. S. A..

[23] Lark DS (2016). Direct real-time quantification of mitochondrial oxidative phosphorylation efficiency in permeabilized skeletal muscle myofibers. Am. J. Physiol. Cell Physiol..

[24] Toime LJ, Brand MD (2010). Uncoupling protein-3 lowers reactive oxygen species production in isolated mitochondria. Free Radic. Biol. Med..

[25] Mailloux RJ (2011). Glutathionylation acts as a control switch for uncoupling proteins UCP2 and UCP3. J. Biol. Chem..

[26] Hempel M (2017). LYRM7 - associated complex III deficiency: a clinical, molecular genetic, MR tomographic, and biochemical study. Mitochondrion.

[27] Walheim E, Wiśniewski JR, Jastroch M (2018). Respiromics: an integrative analysis linking mitochondrial bioenergetics to molecular signatures. Mol. Metab..

[28] Lidell ME, Betz MJ, Enerbäck S (2014). Brown adipose tissue and its therapeutic potential. J. Intern. Med..

[29] Iuso A, Repp B, Biagosch C, Terrile C, Prokisch H (2017). Assessing mitochondrial bioenergetics in isolated mitochondria from various mouse tissues using Seahorse XF96 analyzer. Methods Mol. Biol..

[30] Forbes JM, Thorburn DR (2018). Mitochondrial dysfunction in diabetic kidney disease. Nat. Rev. Nephrol..

[31] Goldberg EJ (2019). Tissue-specific characterization of mitochondrial branched-chain keto acid oxidation using a multiplexed assay platform. Biochem. J..

[32] Ruprecht JJ, Kunji ERS (2020). The SLC25 mitochondrial carrier family: structure and mechanism. Trends Biochem. Sci..

[33] Palmieri F, Monné M (2016). Discoveries, metabolic roles and diseases of mitochondrial carriers: a review. Biochim. Biophys. Acta.

[34] McLaughlin KL, McClung JM, Fisher-Wellman KH (2018). Bioenergetic consequences of compromised mitochondrial DNA repair in the mouse heart. Biochem. Biophys. Res. Commun..

[35] Messer JI, Jackman MR, Willis WT (2004). Pyruvate and citric acid cycle carbon requirements in isolated skeletal muscle mitochondria. Am. J. Physiol. Cell Physiol..

[36] Glancy B, Willis WT, Chess DJ, Balaban RS (2013). Effect of calcium on the oxidative phosphorylation cascade in skeletal muscle mitochondria. Biochemistry.

[37] McLaughlin KL, Kew KA, McClung JM, Fisher-Wellman KH (2020). Subcellular proteomics combined with bioenergetic phenotyping reveals protein biomarkers of respiratory insufficiency in the setting of proofreading-deficient mitochondrial polymerase. Sci. Rep..

[38] Calvo SE, Clauser KR, Mootha VK (2016). MitoCarta20: an updated inventory of mammalian mitochondrial proteins. Nucleic Acids Res..

[39] Naugler C, Lesack K (2011). An open-source software program for performing Bonferroni and related corrections for multiple comparisons. J. Pathol. Inform..

[40] Benjamini Y, Hochberg Y (1995). Controlling the false discovery rate: a practical and powerful approach to multiple testing. J. R. Stat. Soc. Ser. B.

[41] Okuda S (2017). JPOSTrepo: an international standard data repository for proteomes. Nucleic Acids Res..

[42] Deutsch EW (2017). The ProteomeXchange consortium in 2017: Supporting the cultural change in proteomics public data deposition. Nucleic Acids Res..

