# Supplementary material for: Novel approach to quantify mitochondrial content and intrinsic bioenergetic efficiency across organs
Source: Sci Rep. 2020 Oct 19;10:17599. doi: 10.1038/s41598-020-74718-1 (PMC7572412; doi:10.1038/s41598-020-74718-1)
Supplement: Supplementary file 1 — Supplementary Information [file 41598_2020_74718_MOESM1_ESM.pdf]

Novel approach to quantify mitochondrial content and intrinsic bioenergetic efficiency across organs.

Kelsey L. McLaughlin[1,3], James T. Hagen[1,3], Hannah S. Coalson[1,3], Margaret A. M. Nelson[1,3], Kimberly A. Kew[2], Ashley R. Wooten[2], and Kelsey H. Fisher-Wellman[1,3,+]

[1]Department of Physiology, [2]Department of Biochemistry and Molecular Biology, East Carolina University, Brody School of Medicine, Greenville, NC 27834, USA. [3]East Carolina Diabetes and Obesity Institute, Greenville, NC 27834, USA.

[+] To whom correspondence should be addressed. E-mail address: fisherwellmank17@ecu.edu

SUPPLEMENTAL FIGURES

Supplement Figure 1.

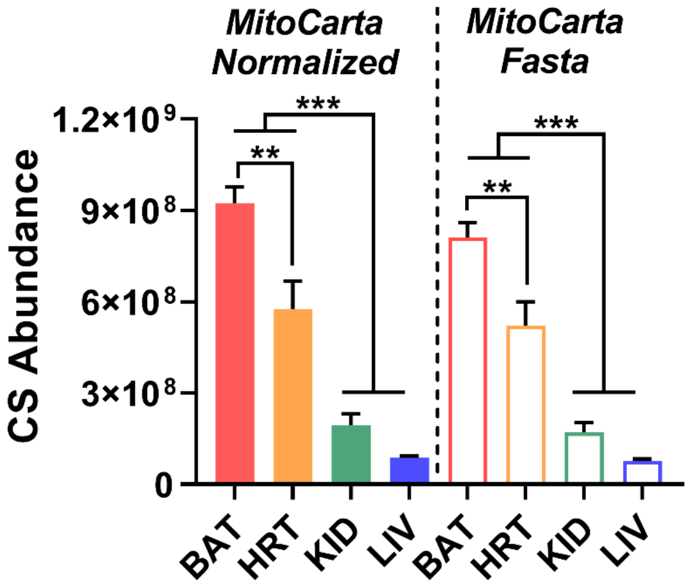

Supplement Figure 1. Comparison of search strategies using the MitoCarta 2.0 database. Citrate synthase (CS) abundance. In the close bars, abundance was determined using the entire mouse proteome database and then results were adjusted for MitoCarta enrichment. The open bars depict MitoCarta 2.0 normalized CS abundance. In this case, the database used for peptide identification was exclusively MitoCarta 2.0. Data are mean ± SEM, N=5, **Adjusted P<0.01, ***Adjusted P<0.001.

SUPPLEMENTAL TABLES

Supplement Table 1 – A. Exported results from PDv2.2. **B.** Analyzed master protein expression and calculation of sample-specific MEF. **C.** MitoCarta 2.0 Database. **D.-G.** Correlations between all identified/quantified mitochondrial proteins and MEF in BAT, HRT, KID, LIV.

Supplement Table 2 – A. Exported results from PDv2.2. **B.** Analyzed mitochondrial master protein expression. **C.** Correlations between all identified/quantified mitochondrial proteins and Pyr/M supported P/O ratio across tissues.

Supplement Table 3 – Statistical comparisons between tissues for each protein listed in figure heatmaps.

Supplement Table 4 – A. Bioenergetic flux data normalized to total protein. **B.** Sample-specific MEF. **C.** Bioenergetic flux data normalized to total protein and corrected for sample-specific MEF.
